# Toxicology of Engineered Nanoparticles: Focus on Poly(amidoamine) Dendrimers

**DOI:** 10.3390/ijerph15020338

**Published:** 2018-02-14

**Authors:** Pratap C. Naha, Sourav P. Mukherjee, Hugh J. Byrne

**Affiliations:** 1 Department of Radiology, Perelman School of Medicine, University of Pennsylvania, Philadelphia, PA-19104, USA; 2 Molecular Toxicology Unit, Institute of Environmental Medicine (IMM), Karolinska Institutet, 17177 Stockholm, Sweden; sourav.mukherjee@ki.se; 3 FOCAS Research Institute, Dublin Institute of Technology, Kevin Street, Dublin 8, Ireland; hugh.byrne@dit.ie

**Keywords:** PAMAM dendrimers, nanotoxicology, engineered nanoparticle, nanomedicine, toxicology

## Abstract

Engineered nanomaterials are increasingly being developed for paints, sunscreens, cosmetics, industrial lubricants, tyres, semiconductor devices, and also for biomedical applications such as in diagnostics, therapeutics, and contrast agents. As a result, nanomaterials are being manufactured, transported, and used in larger and larger quantities, and potential impacts on environmental and human health have been raised. Poly(amidoamine) (PAMAM) dendrimers are specifically suitable for biomedical applications. They are well-defined nanoscale molecules which contain a 2-carbon ethylenediamine core and primary amine groups at the surface. The systematically variable structural architecture and the large internal free volume make these dendrimers an attractive option for drug delivery and other biomedical applications. Due to the wide range of applications, the Organisation for Economic Co-Operation and Development (OECD) have included them in their list of nanoparticles which require toxicological assessment. Thus, the toxicological impact of these PAMAM dendrimers on human health and the environment is a matter of concern. In this review, the potential toxicological impact of PAMAM dendrimers on human health and environment is assessed, highlighting work to date exploring the toxicological effects of PAMAM dendrimers.

## 1. Introduction

Nanotechnology is a new and rapidly emerging field that involves the design, production, and exploitation of structures at the nanoscale. A nanomaterial is a material that has one or more external dimensions in the nanoscale or which is nanostructured. A nano-object with all three external dimensions in the nanoscale is defined as a nanoparticle [1]. Nanotechnology is a sector of the materials manufacturing industry that has already created a multibillion market which is widely expected to grow to 4.4 trillion $US by 2018 [2]. Numerous applications of nanoparticles are already on the market, in products such as paints, sunscreens, cosmetics, self-cleaning glass, industrial lubricants, advanced tyres, semiconductor devices, medicine, and food [3,4,5].

In the biomedical field, nanotechnology is also projected to have a significant impact and has already created a 96.9 billion $US market [4]. For example, polymeric micro and nanoparticles have been proposed for a wide range of medical applications in terms of diagnosis, tissue engineering, and as drug delivery devices [6,7,8,9,10,11,12]. Several nano-formulations like Doxil (liposome-based nanoparticles), DaunoXome (PEGylated lipososmal doxorubicin), Myocet (Liposomal daunorubicin), Abraxane (albumin-bound paclitaxel nanoparticles) have been approved for clinical use and several other nanoformulations are undergoing clinical trials [13]. Inorganic nanomaterials are being proposed as imaging probes for magnetic resonance imaging (MRI) [14,15], computed tomography (CT) [16,17], and dual-energy mammography (DEM) [18,19]. Polymeric nanomaterials based on US Food and Drug Administration (FDA) approved polymers such as poly(lactic-co-glycolic acid) (PLGA) and poly(lactic acid) (PLA) have been proposed as drug delivery vehicles and vaccine delivery systems [20,21,22,23] and for tissue engineering [24]. 

Dendritic nanoparticles such as poly(amidoamine) (PAMAM) dendrimers have already been explored for applications such as targeted drug delivery in cancer therapy [25,26,27,28], gene delivery [29,30,31], medical imaging applications [32,33,34,35], and also the mannosylated form of PAMAM dendrimers has been proposed for vaccine delivery systems [36].

## 2. Toxicology of Engineered Nanoparticles

Nanotoxicology is an evolving sub-specialty of particle toxicology. It addresses the toxicology of nanoparticles, which in general appear to elicit specific biological responses that are governed by their nano-size which absent in bulk form. The proliferation of nanotechnology, as well as increase in the anthropogenic nanoparticulate matter has prompted concerns over the safety of these nano-sized particles per se, that may have either intentional or occupational exposure to human or the environment. Therefore, it is important to understand the interaction of nanomaterials with living organisms in terms of potential toxicological impacts on both the environment and human health.

Nanotoxicology was mostly focused on individual studies, with one to a few nanomaterials, on the biological interaction of nanoparticles without greater integration and coherence among them [37,38,39,40,41,42,43,44,45,46]. However, more recently bigger consortia have been formed, e.g., EU sponsored FP7-NANOREG project etc., with integrated and harmonised approaches for screening of a wide range of nanomaterials in parallel with different biological models using standardised protocols [47]. Physico-chemical characteristics of the nanoparticles such as particle size, surface area, surface charge and morphology have been identified as important factors in determining their toxic effects [39,45,48,49,50] and the importance of appropriate characterisation of these properties has been highlighted [51]. The smaller a particle is, the larger its surface area to volume ratio is, and thus the associated chemical and biological activity of the material is increased. The greater chemical reactivity of nanomaterials results, for example, in increased production of reactive oxygen species (ROS) [50,52], including free radicals. In the case of nanotoxicology, increased levels of intracellular ROS as a result of nanoparticle exposure have been identified as a fundamental precursor to inflammation, genotoxicity, and apoptosis [53]. Oxidative stress induces signaling pathways of MAPK and transcription factors such as NFkB, AP-1 [54,55,56]. These transcription factors induce mRNA expression of pro-inflammatory mediators, finally causing inflammation. Persistent inflammation can lead to cell damage, induced by chemical/physical injury, anoxia, or nanoparticles. When nanoparticles enter into the bloodstream, they immediately encounter a complex environment of plasma proteins and immune cells [57]. In addition, some nanoparticles seem to be able to translocate from their site of deposition to more remote sites such as brain [58,59]. Notably, toxicity of the engineered nanoparticles is dependent upon the surface coating materials; in a recent study of gold nanoparticles, it was observed that the in vitro toxicity was due to the coating materials rather than exposure dose or time [60].

In addition to potential human hazards, the effect of nanoparticles on the environment should also be considered. The assessment of adverse environmental effects of nanoparticles requires a detailed understanding, for example, mobility, reactivity, toxicity, and bio-persistence in the environment [61]. Over the last decade, an increasing number of ecotoxicological studies of engineered nanomaterials have emerged in the literature. Many studies have focused on carbon based material [62,63,64,65,66,67,68], various metallic nanoparticles (e.g., silver, zinc, cadmium, iron oxide, etc.) [69,70,71], as well as polymeric nanoparticles (e.g., dendrimers) [48,49,72]. Carbon based materials, such as carbon nanotubes, have been investigated in a wide range of ecological organisms, such as algae [73], daphnia [74], and fish [75]. Ecotoxicity of metallic nanoparticles, such as silver nanoparticles, have been also been investigated [76,77,78,79]. Oprsal et al. reported that the toxicity of silver nanoparticles was due to bioaccumulation of sedimented silver nanoparticle aggregates in the fish model and therefore an increase in the local silver concentration can be responsible for the toxicity [79]. Ramskov et al., reported that, in invertebrate sediment dwelling organism, the bioaccumulation was much higher in worms than in snails, which can be due to sediment avoidance behavior of the snail species [78]. However, the toxicity, in terms of growth and mortality, of silver nanoparticles also differed among the worm species used in this study. Such a species-specific effect is likely related to differences in uptake route, internalization, and detoxification capacity.

## 3. PAMAM Dendrimers

PAMAM dendrimers are well-defined nanoscale constructs, originally synthesised by Tomalia et al., and have a range of potential novel applications in the biomedical field [80]. PAMAM dendrimers contain a 2-carbon ethylenediamine core and for each increase of generation, by a stepwise synthesis, the effective surface charge, molecular weight, and size increases systematically. With increasing generation, the number of surface amine groups increases, while the distance between the surface charges decreases, which helps to seal the interior from the bulk solution [81]. These properties can be useful to enhance the encapsulation and release of different drugs from the dendrimers. The quantity of entrapped guest molecules depends upon their size and shape, as well as size of the dendrimer’s internal cavities. It has been proposed that these dendrimers can be opened under controlled conditions to release entrapped drug molecules by partial or total hydrolysis [82,83,84]. Thus, the systematically variable structural architecture and the large internal free volume make these dendrimers an attractive option for drug delivery and other biomedical applications [85,86,87,88]. 

Kannan et al. have reviewed the development of dendrimer based applications in nanomedicine, describing the evolution of polymer based nanomedicine as well as the challenges to advancing nanomedicine/therapies into clinical trials and legislative approval. In terms of critical nanoscale design parameters, they advocate the use of dendritic nanoparticles as their functions involving PKs, PDs, drug delivery strategies, excretion modes, biodistribution patterns, biocompatibility, and nanotoxicology can readily be optimised, and describe the state of the art of dendrimer-based in vivo therapies [89].

It is also possible to passively target PAMAM dendrimers to a tumor because of the increased permeability of tumor vasculature to macromolecules and also due to the limited lymphatic drainage [90]. The unique properties of dendrimers, as compared to linear polymers, render them of interest for intracellular drug delivery system for cancer therapy [91]. PAMAM dendrimers have also been proposed for tumor targeting using FDA-approved antibodies, such as trastuzumab [92,93,94] and cetuximab [95,96], against epidermal growth factors. 

Amine terminated PAMAM dendrimers have been shown to enhance anti-ovalbumin immunoglobulin-G and immunoglobulin-M levels and have also been used as adjuvants in vaccine delivery systems [97]. *N*-acetyl-d-glucosamine modified PAMAM dendrimers improve the immunogenicity by upregulation of antibody formation via activation of natural killer cells [98]. Another study showed that PAMAM dendrimers of the mannosylated form potentiate the immunogenicity and they have been proposed for vaccine delivery systems [36]. 

PAMAM dendrimers are also well documented for the application in magnetic resonance imaging (MRI) using Gd-radiolabeled PAMAM dendrimers with DTPA chelate [99]. Using PAMAM dendrimers as a vehicle, Gd-DTPA-PAMAM dendrimers increase the longitudinal relaxation (r2 relaxivity) which improves the MR signal in the surrounding tissue. Also, it has been reported that conjugation of Gd-DTPA with PAMAM dendrimers improves the stability of the Gd-DTPA complex and reduces renal toxicity due to free gadolinium, which was observed in free Gd-DTPA (Magnevist^®^). It has also been shown that folate-conjugated PAMAM-Gd significantly improves the tumor targeting and increases the MR signal in the tumor [100].

**Figure 1:**
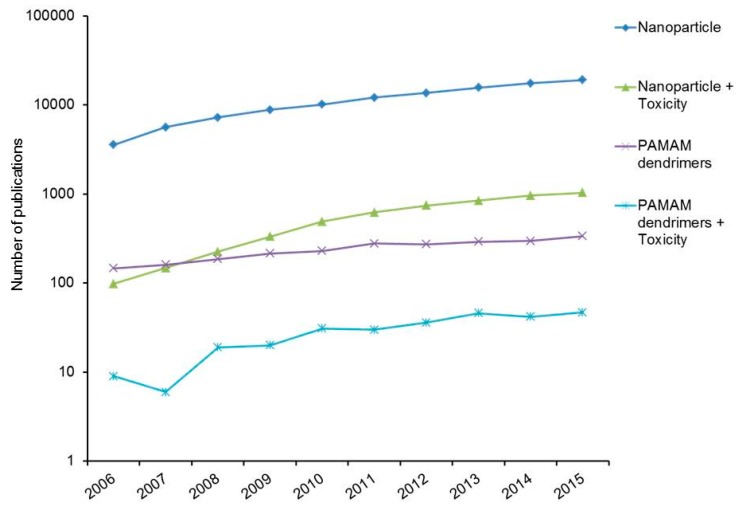
Number of publication on nanoparticle, nanoparticle toxicity, PAMAM dendrimers, and PAMAM dendrimer toxicity. Data acquired from the Web of Science database.

Notably, however, in recent times, the number of articles on the topic of nanoparticle toxicity, PAMAM dendrimers, and PAMAM dendrimer toxicity published and cited has been increasing daily (Figure 1). While much of the published research has focused on in vitro or in vivo toxicity of nanoparticles, while studies of environmental impacts, or ecotoxicity, have only more recently begun to emerge.

### 3.1. In Vitro Toxicological Studies of PAMAM Dendrimers 

#### 3.1.1. In Vitro Mammalian Toxicological Studies

The wide range of proposed applications of PAMAM dendrimers merit an assessment of the environmental health risk and indeed the OECD has identified that there is an urgent need of study of the human and environmental related toxicity of dendritic polymer nanoparticles [101]. Cytotoxicological assessment of the PAMAM dendrimers (G4, G5, and G6) has indicated adverse effects to mammalian cells in vitro in a dose dependent manner [45,72,102,103,104]. A recent study shows that the pathway of the toxic response induced by PAMAM dendrimers is by apoptosis mediated by mitochondrial dysfunction [105]. Studies by other groups reported that PAMAM dendrimers contribute to reduction of transmembrane potential and hinder the influx of Ca^2+^ ions to the mitochondria [106] and PAMAM dendrimers have been shown to induce membrane disruption, including formation of holes and membrane erosion in supported lipid bilayaers [107,108,109]. The toxicity of PAMAM dendrimers in mammalian cells has been demonstrated to depend upon the generation and number of surface functional groups [45,49,72,102,103,104,110,111,112]. The positively charged surface functional groups of PAMAM dendrimers are responsible for the toxicity and destabilise the cell membrane and cause cell lysis [113]. Although systematic studies of the generation dependent toxicity extending to higher and lower generation number have not been reported, systematic studies of the structurally related poly(propylene imine) (PPI) dendrimers indicated that the lower generations (G0–G2) are uptaken passively rather than actively and are non-toxic to mammalian cells, indicating the importance of the cellular uptake mechanism [114]. 

**Figure 2:**
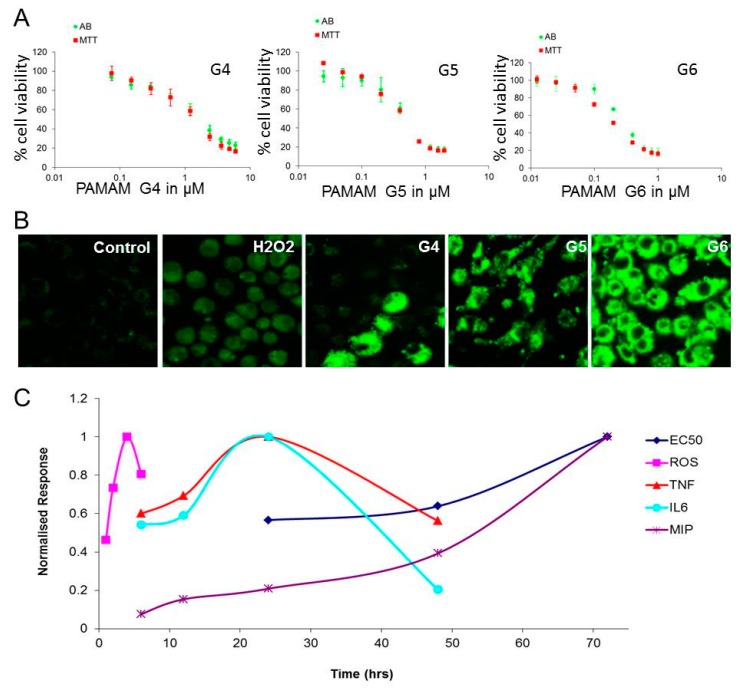
Panel (**A**), dose depend cytotoxicity effect of PAMAM dendrimer in J774A.1; cytotoxicity assay was performed via alamar blue (AB) and MTT assay. The concentration ranges used for the cytotoxicity assays with G4, G5, and G6 were 0.08 μM to 6 μM; 0.03 μM to 2 μM; and 0.013 μM to 1 μM respectively, as determined from an initial range finding study. Panel (**B**), Confocal laser scanning micrographs of intracellular ROS generation in J774A.1 cells, following exposure to negative control, positive control (H_2_O_2_), PAMAM G-4, PAMAM G-5, and PAMAM G-6. The data are shown after a 2 h exposure of PAMAM dendrimers. Panel (**C**), graphical representation of different responses as a function of time. Figure reproduced with permission from reference [45].

In mouse macrophage cells, it was observed that PAMAM dendrimers (G4, G5, and G6) produce a dose and generation dependent cytotoxicity and the toxicity correlated with the number of surface amine groups per generation [42]. The mechanism of the toxicity is due to the generation of intracellular reactive oxygen species (ROS) which leads to a cascade of secretion of pro-inflammatory markers and finally cell death (Figure 2). In human skin and intestinal cells, the toxicity of PAMAM dendrimers was also seen to be dependent upon the exposure dose and generation of PAMAM dendrimers using four different assays, MTT, alamar blue, neutral red, and the clonogenic assay [103]. Following preliminary cytotoxicity investigation, the toxicity mechanism of PAMAM dendrimers was explored and it was observed that PAMAM dendrimers are localised in the early endosomes/lysosomes but can then migrate to mitochondria upon lysis of the endosome/lysosomes [105]. A biphasic ROS generation profile was observed, the earlier associated with the localisation in the endosomes, the later in the mitochondria. The mechanisms of subsequent cellular responses leading to inflammatory responses, apoptosis, and cell death have been proposed and modelled using a phenomenological rate equation approach [45,72,102,115]. A comparative toxicity study of PAMAM and lipid-PAMAM was reported recently by Bertero et al., wherein they showed that both the dendrimer forms are able to enter into endothelial and primary neural cells. However, only the PAMAM dendrimers induced a toxic response [116]. Another study of PAMAM G4 and G4-C12 modified PAMAM dendrimers was carried out in primary neural cells. G4-C12 modified PAMAM (at 100 nM concentration) has an adverse effect on the primary neural cell in vitro, whereas PAMAM G4 does not induce apoptotic cell death at sub-micromolar concentrations [117]. Cationic PAMAM dendrimers have been reported to cause cell cycle arrest in primary human lung cell line at low (noncytotoxic) dose, but not in lung-derived cancer cells [118]. Transcriptomics analysis andβ-galactosidase staining suggested that PAMAM at this low dose also induced senescence in the primary lung cells. In contrast, hydroxyl functionalised PAMAM dendrimers did not show any such effect on cell cycle progression in this study.

#### 3.1.2. Physicochemical Properties of PAMAM Dendrimers versus Toxicity

The surface coating has been seen to determine the cytotoxicity/biocompatibility for many nanoparticles [119]. In the case of PAMAM dendrimers, the toxicity is largely due to the surface amine groups, and several studies have reported that after surface modification (resulting in neutral or anionic surfaces) the toxicity of PAMAM dendrimers can be reduced. A number of studies have explored the underlying mechanisms of this dependence of the toxic response on dendrimer surface properties.

G2 and G3 of the cationic phosphorous dendrimer have been tested in murine embryonic hippocampal cells (mHippoE-18) and both dendrimers induce mHippoE-18 cell death by generation of reactive oxygen species, alteration of the mitochondrial membrane potential, changes in cell cycle phase, and DNA damage [120]. Cationic PAMAM dendrimers are haemolytic and cytotoxic, depending on the molecular weight, number of surface amine groups, and generation of PAMAM dendrimers; whereas anionic PAMAM dendrimers and PEO modified CSi-PEO dendrimers are neither haemolytic nor cytotoxic [110]. Another study demonstrated the effect of surface functionality of PAMAM dendrimers on enzyme activity. In this study, cationic (G4 PAMAM –NH_2_), neutral (G4 PAMAM –OH), and carboxylated (G3.5 PAMAM –COOH) dendrimers were employed. It was seen that positively and neutral charged PAMAM dendrimers inhibit the enzyme activity of pepsin, whereas negatively charge PAMAM dendrimers have no effect of enzyme inhibition [121]. Surface modification of G5 PAMAM dendrimer with zwitterionic carboxybetamine was seen to reduce the toxic effect of dendrimers [122]. Similar surface modification of G4 PAMAM dendrimer with 4-carbomethoxypyrrolidone reduced the toxicity in terms of intracellular ROS generation and alteration of mitochondrial membrane potential [123]. When the surface of PAMAM G4 is modified with dimethyl itaconate, resulting in pyrrolidone at the PAMAM surface, it was found that no haemolytic effect was observed [124]. PEGylation also dramatically reduced the haemolytic effect of PAMAM dendrimers [113] and another study reported that systematic replacement of the surface amine groups with acetyl groups in PAMAM dendrimers resulted in a 10-fold reduction of the toxicity [125].

#### 3.1.3 Cellular Uptake and Biodegradation

Due to smaller size of nanoparticles, including dendrimers, they are likely to use a range of cellular machinery for internalisation and trafficking to various sub-cellular compartments. The pH also varies in different subcellular compartments of the cell, as well as, in different organs. The pH becomes more acidic successively from endocytic vesicles to early endosomes (pH 6.5), late endosomes, and lysosomes (pH < 5.5) [126]. Seib et al. have shown that PAMAM can enter the cells by endocytosis [127], while Lee et al. reported that the configuration of dendrimers is dependent on the pH of the surrounding [128]. Differences in cellular uptake mechanisms may exist depending on the surface properties. For instance, in A549 lung epithelial cells (a cancer cell line) anionic G3.5-carboxyl-terminated PAMAM were shown to mainly be taken up by caveolae-mediated endocytosis, whereas hydroxyl and amine terminated G4 PAMAM were taken up by a non-clathrin-dependent mechanism [129]. Albertazzi et al. demonstrated that dendrimers are internalised by both clathrin-dependent endocytosis and macropinocytosis in HeLa cells and are eventually delivered to the lysosomal compartment [130]. Overall, understanding the stability of dendrimers at different pH values is important as this will aid in the understanding of their intracellular fate and, hence, in their utility as drug delivery vehicles or as imaging probes. The biocompatibility of dendrimers must be accompanied by a fast renal elimination, excretion, or by biodegradation, while the degradation products must be non-toxic [80,131]. Commercially available PAMAM dendrimers are reported to be relatively resistant to hydrolysis [132]. Feliu et al. reported that hydroxyl terminated 2,2-bis(methylol)propionic acid dendrimers (bis-MPA) are degradable at physiological pH (7.5) but not at acidic pH (4.5) (no degradation after 40 days), indicating the stability increases at low pH. These dendrimers were also found to be non-cytotoxic to HeLa, THP.1 cell lines and also to primary human macrophages [131]. 

Recent studies have demonstrated that, when immortalised, non-cancerous human keratinocyte (HaCaT) cells were treated with DL-Buthionine-(S,R)-sulfoximine (BSO), the active uptake of PAMAM dendrimers was suppressed, and when passively uptaken, the dendrimers act as anti-oxidants, rather than oxidants, eliminating the initial phase of endocytosis associated oxidative stress contribution to the toxic response [133]. BSO has previously been employed to study the effects of the reduction of levels of the intracellular antioxidant glutathione (GSH); and therefore oxidative stress [134,135,136,137], but has also been shown to cause membrane permeabilisation. The increased permeability of the membrane allows passive uptake of the dendrimers, by passing the endocytosis pathway [138]. Notably, in a study by Khalid et al. while the larger, higher generations of the aminated nanoscale dendrimers poly(propylene imine) (PPI) were demonstrated to elicit oxidative stress and significant toxicity, the smaller, lower generations exhibited intracellular antioxidant behaviour and low toxicity [114]. Importantly, these studies highlight that the process of endosomal digestion of the dendrimers in itself contributes significantly to the toxic response. As a strategy for drug release, endosomolysis can be extremely disruptive to the cell [102] and therefore, in the case of cationic nanoparticles for intracellular nanomedical applications, avoiding the process of endocytosis may be a valid strategy to pursue [139]. In terms of therapeutic applications, direct entry into the cytosol may be a more convenient route for drug or gene delivery.

#### 3.1.4. In Vitro Eco Toxicological Studies

As well as potential hazards of human exposure, the increased use of dendrimers in a range of nanomedical applications increases the risks of environmental exposure, warranting a thorough investigation of potential ecotoxicological impacts of these materials. Although such studies are limited, the results are consistent with those observed for human based studies.

**Figure 3:**
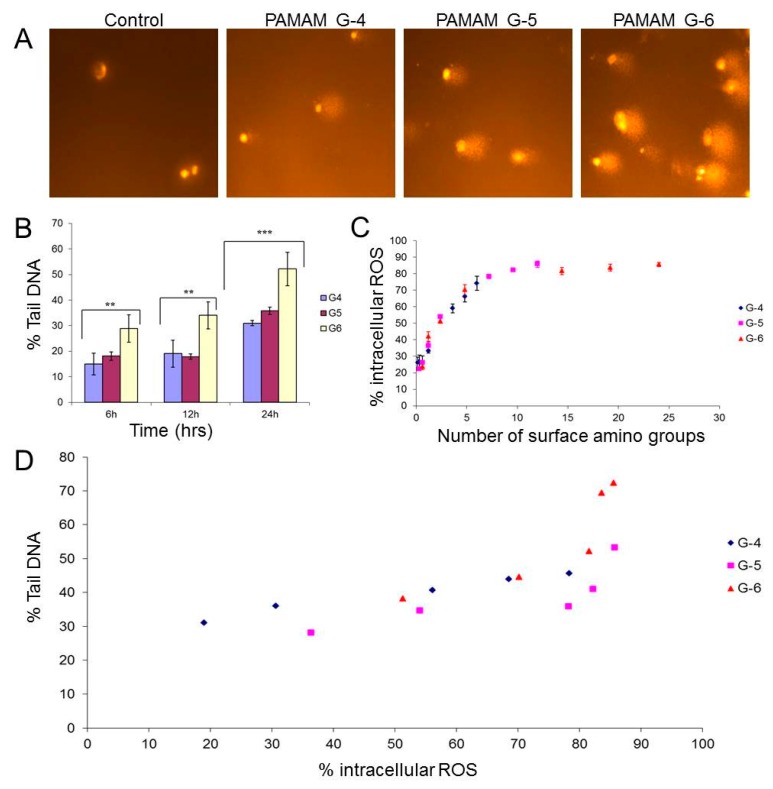
(**A**) Representative micrographs showing comets of PLHC-1 cells after 24 h exposure to PAMAM dendrimers (G4, G5, and G6) at a dose of 0.5 µM concentration. (**B**) Generation dependent genotoxicity response of PAMAM dendrimers at 6, 12, and 24 h exposure at 0.5 µM concentration, as represented by the %Tail DNA measured by the comet assay. *** Significant difference of genotoxicity response between G4, G5 and G6 (*p* ≤ 0.05). ** Significant difference of genotoxicity response between G4, G6, and between G5, G6 (*p* ≤ 0.05). (**C**) Plot showing the relationship between the intracellular ROS production and the molar volume X number of surface amine groups at 2 h exposure time period of 0.6 µM concentration. (**D**) Relationship between DNA damage and intracellular ROS production upon exposure to PAMAM dendrimers G4, G5, and G6. Maximum DNA damage was observed at 24 h, and maximum ROS at 2 h. Figure reproduced with permission from reference [72].

As in the case of in vitro cytotoxicological studies in mammalian systems, a systematic dose and generation dependent toxicological response was observed in fresh water ecological organisms [49]. Rainbow trout gonadal fish cell-line (RTG-2) was seen to be the least sensitive to the PAMAM dendrimers [49]. Toxicity was found to be generation dependent in each test organism; the toxicity order is G6 > G5 > G4. Among the two fish cell lines used, PLHC-1 (*Poeciliopsis lucida* hepatocarcinoma cells) is more sensitive than RTG-2. Furthermore, the toxic mechanism of PAMAM dendrimers was investigated in the fish cell line (PLHC-1) by Naha et al. and the results suggested that the toxicity is initiated by the generation of reactive oxygen species, followed by DNA damage and cell death (Figure 3) [72]. The generation of ROS and DNA damage is related to the number of surface amine group and dendrimer generation [72]. As the envisioned applications of PAMAM dendrimers are in low dose nanomedical applications, it is expected that the release the environment through domestic or even clinical waste will be very low. However, a greater potential exposure risk may be presented through industrial effluent from the source manufacturers, and so a risk management strategy should be established.

### 3.2. In Vivo Toxicological Studies of PAMAM Dendrimers

In vivo toxicity of PAMAM dendrimers has barely been explored. In this section, the potential adverse effects of PAMAM dendrimers demonstrated in different test models (zebrafish, mice, rat, rabbit model, etc.) are highlighted.

**Figure 4:**
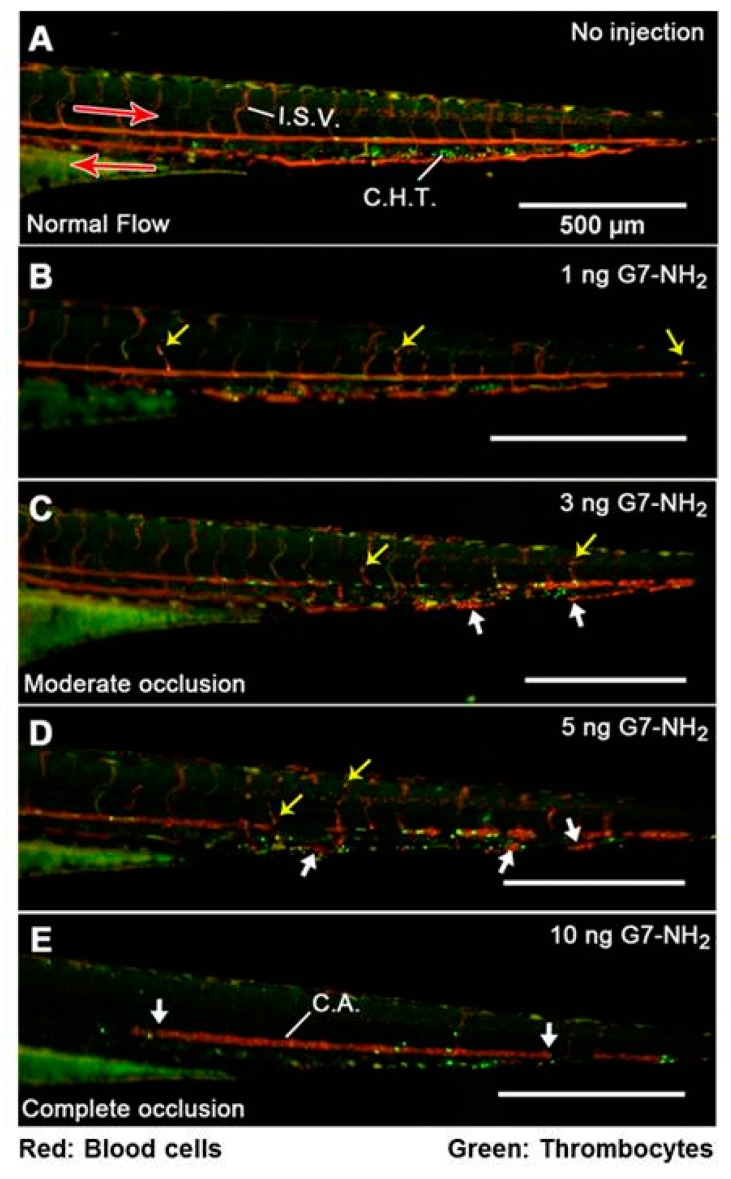
Dose-dependent G7-NH_2_ occlusion in zebrafish embryos 1 min post injection. The top panel (**A**) depicts unperturbed caudal blood flow as streaks of red and green light visible in the major caudal artery (CA, left to right), vein (right to left), and intersegmental vessels. Punctate green and red spots in between the major caudal artery and vein indicate the location of the caudal hematopoietic tissue. Subsequent images (proceeding downward (**B**–**E**), with increasing G7-NH_2_ dose) depict increased cellular adhesion and vascular occlusion resulting from dendrimer injection, culminating in no visible blood flow following an injection of 10 ng of G7-NH_2_. The yellow arrows depict individual red blood cells trapped inside the vessel, while the large white arrows show significant blood clots inside the vessels. Notice in (**E**) that the caudal artery is completely occluded with no observable flow in the major caudal vein. Figure reproduced with permission from reference [140].

Recently, an in vivo study claimed that cationic dendrimers induced fibrinogen aggregates and formation of clots in blood vessels in a zebrafish model when injected intravenously (Figure 4). In this study, PAMAM G7 was injected intravenously into zebrafish, and it was found that PAMAM G7 dendrimers induce aggregation of many blood proteins, such as fibrinogen, albumin, etc. Most of the blood proteins have negative surface charge, and therefore cationic dendrimers aggressively interact with them and form blood clots (Figure 4) [140]. A further study in a rodent model showed the formation of blood clots after intravenous injection of PAMAM dendrimers [141]. Therefore, intravenous administration of these cationic dendrimers is not safe for therapeutic applications. Surface modification is imperative to make the dendrimer biocompatible [87,123,124,142]. 

Another study in a mouse model showed that PAMAM dendrimer G-5 can cause acute lung failure when administered via the intranasal route. The mechanism of this adverse effect is via binding of the PAMAM dendrimer to the angiotensin-converting enzyme 2, downregulating its function and expression in lung tissue. This results in deregulation of the renin-angiotensin system [143]. Furthermore, a study on PAMAM G4 and G4-C12 modified PAMAM dendrimers in a mouse model showed that both dendrimer forms are able to penetrate into neurons after intra-ventricular injection. PAMAM G4 does not induce apoptotic cell death at sub-micromolar concentrations, but induces low microglia activation in the brain tissue after a week [117]. A recent study by Durocher and Girard has shown pro-inflammatory activities of PAMAM dendrimers (G0–G3) in vivo using a murine air pouch model. They found that PAMAM dendrimers rapidly increased leukocyte influx after 3 h, the vast majority of cells being neutrophils. In addition, they observed the production of several cytokines/chemokines. The pro-inflammatory activities were correlated with the dendrimer generation, i.e., G3 > G2 > G1 > G0 [144]. 

**Figure 5:**
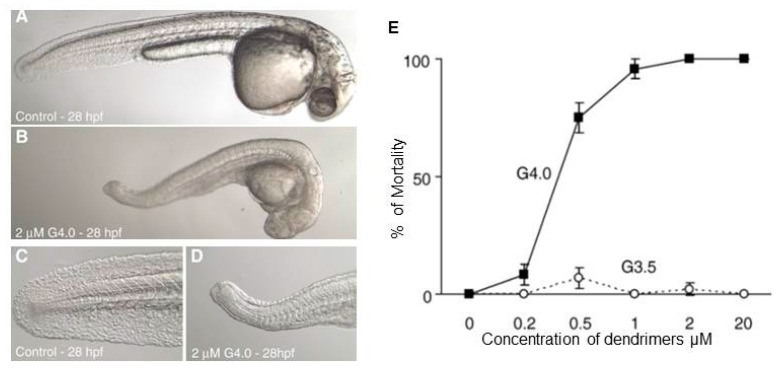
Representative micrographs of overt toxicity seen in zebrafish embryos exposed to control or 2 μM G4 dendrimers beginning at 6 h post fertilization (hpf). All micrographs were taken at 28 hpf. (**A**) Control embryo manually removed from chorion (magnification 3.2×). (**B**) G4 dendrimer-treated embryo manually removed from chorion (3.2×). (**C**) Higher magnification of tail of control embryo (10×). (**D**) Higher magnification of tail of G4 dendrimer-treated embryo (10×). (**E**) Mortality of zebrafish embryos at 120 hpf following exposure to G4 and G3.5 PAMAM dendrimers from 6 to 120 h post fertilization (hpf). Figure reproduced with permission from [145].

Notably, an interesting study by Heiden et al. reported that amine terminated PAMAM dendrimers adversely affect the growth and development of zebrafish embryos (Figure 5) at sub-lethal concentrations [145]. In this study, PAMAM G-4 and G-3.5 were investigated, and the results suggested that cationic PAMAM dendrimers significantly inhibit the development of zebrafish embryos compared to anionic PAMAM dendrimers. Both in vitro and in vivo toxicity studies on PAMAM dendrimers revealed that amine terminated PAMAM dendrimers produce higher levels of toxicity than hydroxyl or carboxylic acid terminated PAMAM dendrimers. Surface modification can therefore enhance the biocompatibility of PAMAM dendrimers. Another interesting study by Bodewein et al. reported the toxic effect of PAMAM and PPI dendrimers in a zebrafish embryo model. They did not observe zebrafish embryo toxicity with anionic PAMAM dendrimers, although exposure to doses greater than 50 µM caused adverse effects to aquatic organisms [112]. 

Biodistribution studies are essential to understand and explore the organ specific toxicity effect of PAMAM dendrimers. A study in a rabbit model reported the biodistribution of PAMAM dendrimers (hydroxyl terminated generation 4), and the results suggested that a significant amount of PAMAM dendrimers is accumulated in the liver, lungs, kidney, and heart. Some dendrimers were also found in the brain of neonatal rabbit with cerebral palsy [146], whereas no PAMAM dendrimers were found in the brain of healthy rabbit. Another study in a mouse model reported the oral bioavailability of PAMAM dendrimer G-6.5, and the results demonstrated that, after oral administration, a substantial amount of PAMAM dendrimers was found in the heart, lungs, liver, blood, urine, stomach, and small and large intestine. The presence of PAMAM dendrimers in blood, liver, and urine suggested that PAMAM G-6.5 crosses the intestinal barrier and reaches the systemic circulation, hence enhancing the oral bioavailability [147]. Organ specific toxicity studies on PAMAM dendrimers have been barely explored, and further more in-depth histopathological studies on each organ need to be undertaken, due to the safety concerns.

## 4. Conclusions

PAMAM dendrimers are well-defined nanomaterials of great interest for biomedical applications such as drug delivery system, in diagnostics and in imaging. In this review, the current information of the PAMAM dendrimers toxicity in different models has been detailed. In summary, amine terminated PAMAM dendrimers induce toxicity in the range of models discussed above. Furthermore, the toxic response increases systematically with generation, correlated with the number of surface amine groups. However, hydroxyl (–OH) and carboxylic acid (–COOH) terminated PAMAM dendrimers have been shown to be significantly less toxic. The importance of surface modification is highlighted, which improves the biocompatibility of the amine terminated PAMAM dendrimers. This is beneficial for the design of biocompatible dendrimers for biomedical applications. However, the pharmacokinetics, biodistribution, biodegradation, and chronic toxicity of PAMAM dendrimers are not yet clearly understood. Further studies in this area are sorely needed for the development of biocompatible dendrimers for biomedical applications.
